# Supplementary material for: Probability of Starting Two-Drug Regimen (2DR) vs. Three-Drug Regimen (3DR) in ART-Naïve and ART-Experienced Person with HIV (PWH) Across the First Wave of COVID-19 Pandemic
Source: Viruses. 2024 Nov 23;16(12):1822. doi: 10.3390/v16121822 (PMC11680257; doi:10.3390/v16121822)

Supplementary material.

Supplementary Table S1.
General characteristics of study population (naïve PWH) according to calendar year of ART initiation

	**Calendar period of ART initiation**				
**Characteristics**	**2019**<br>**N= 870**	**2020**<br>**N= 522**	**2021-2022**<br>**N= 1089**	**p-value***	**Total**<br>**N=2481**
**Female, n (%)**	157 (18.0)	92 (17.6)	175 (16.1)	0.481	424 (17.1)
**Age, years, median (IQR)**	40 (31, 50)	40 (31, 51)	41 (33, 51)	0.188	40 (32, 51)
**Mode of HIV Transmission, n(%)**					
**IDU**	58 (6.7)	21 (4.0)	64 (5.9)	0.054	143 (5.8)
**Homosexual contacts**	398 (45.7)	248 (47.5)	515 (47.3)		1161 (46.8)
**Heterosexual contacts**	358 (41.1)	208 (39.8)	405 (37.2)		971 (39.1)
**Other/Unknown**	56 (6.4)	45 (8.6)	105 (9.6)		206 (8.3)
**Not Italian, n (%)**	248 (28.5)	160 (30.7)	305 (28.0)	0.538	713 (28.7)
**Calendar year of baseline, median (IQR)**	2019 (2019, 2019)	2020 (2020, 2020)	2021 (2021, 2022)	<.001	2020 (2019, 2021)
**AIDS diagnosis, n(%)**	98 (11.3)	46 (8.8)	112 (10.3)	0.346	256 (10.3)
**CD4 count, cells/mmc, <=200 cells/mm³**	300 (34.8)	163 (31.4)	365 (34.1)	0.423	828 (33.8)
**HIV RNA >100,000 copies/mL, n(%)**	171 (19.7)	105 (20.2)	267 (24.5)	0.020	543 (21.9)
**Site geographical position, n(%)**				0.003	
**North**	433 (49.8)	247 (47.3)	608 (55.8)		1288 (51.9)
**Central**	343 (39.4)	224 (42.9)	401 (36.8)		968 (39.0)
**South**	94 (10.8)	51 (9.8)	80 (7.3)		225 (9.1)
**eGFR (CKD_epi formula), ml/min/1.73m², median, (IQR)**	106.5 (94.60, 117.2)	106.2 (93.92, 116.9)	105.1 (92.87, 115.5)	0.155	106.2 (93.74, 116.4)
**Diabetes, n(%)**	20 (2.3)	15 (2.9)	15 (1.4)	0.103	50 (2.0)

**Smoking, n(%)**	252 (29.0)	152 (29.1)	297 (27.3)	0.622	701 (28.3)
**Total cholesterol, mg/dL, median, (IQR)**	158 (136, 184)	159 (137, 187)	159 (135, 183)	0.812	159 (136, 18)
**Use of statins, n(%)**	16 (1.8)	6 (1.1)	14 (1.3)	0.483	36 (1.5)
**Use of antihypertensive drugs, n(%)**	41 (4.7)	23 (4.4)	53 (4.9)	0.920	117 (4.7)

Abbreviations: IDU, intravenous drug user; eGFR, estimated Glomerula Filtration Rate; CKD_epi, Chronic Kidney Disease Epidemiology Collaboration

**Chi-square or Kruskal-Wallis test as appropriate*

Supplementary Table S2
General characteristics of study population (ART-experienced virologically suppressed PWH) by calendar year of ART switch

	Calendar period of ART switch				
**Characteristics**	**2016-2018 N= 7082**	**2019-2020 N= 3306**	**2021-2022 N= 1947**	**p-value***	**Total N= 12,335**
**Female, n (%)**	1454 (20.5)	680 (20.6)	381 (19.6)	0.619	2515 (20.4)
**Age, years, median (IQR)**	46 (38, 54)	47 (38, 55)	48 (39, 57)	<.001	47 (38, 55)
**Mode of HIV Transmission, n(%)**					
IDU	757 (10.7)	273 (8.3)	154 (7.9)	<.001	1184 (9.6)
Homosexual contacts	3115 (44.0)	1593 (48.2)	912 (46.8)		5620 (45.6)
Heterosexual contacts	2806 (39.6)	1258 (38.1)	764 (39.2)		4828 (39.1)
Other/Unknown	404 (5.7)	182 (5.5)	117 (6.0)		703 (5.7)
**Not Italian, n (%)**	1083 (15.3)	560 (16.9)	277 (14.2)	0.020	1920 (15.6)
**Calendar year of switch, median (IQR)**	2017 (2017, 2018)	2019 (2019, 2020)	2021 (2021, 2022)	<.001	2018 (2017, 2020)
**AIDS diagnosis, n(%)**	1036 (14.6)	501 (15.2)	277 (14.2)	0.631	1814 (14.7)
**CD4 count nadir, cells/mmc, median (IQR)**	281 (150, 415)	294 (154, 440)	299 (155, 437)	<.001	287 (152, 425)
**CD4 count, cells/mmc, median (IQR)**	680 (487, 893)	700 (514, 927)	744 (554, 964)	<.001	696 (505, 915)
**CD4 count, cells/mmc, <=200 cells/mm^3**	243 (3.4)	81 (2.5)	41 (2.1)	0.001	365 (3.0)
**Site geographical position, n(%)**				<.001	
North	4068 (57.4)	1869 (56.5)	1147 (58.9)		7084 (57.4)
Center	2510 (35.4)	1222 (37.0)	551 (28.3)		4283 (34.7)
South	504 (7.1)	215 (6.5)	249 (12.8)		968 (7.8)
**eGFR (CKD_epi formula), ml/min/1.73m^2, median, (IQR)**	89.01 (75.46, 101.5	84.74 (72.10, 98.03)	80.93 (69.36, 93.78)	<.001	86.52 (73.47, 99.64)
**Diabetes, n(%)**	379 (5.4)	162 (4.9)	108 (5.5)	0.522	649 (5.3)
**Smoking, n(%)**	2820 (39.8)	1196 (36.2)	717 (36.8)	<.001	4733 (38.4)

**Total cholesterol, mg/dL, median, (IQR)**	182 (156, 209)	190 (165, 216)	191 (163, 217)	<.001	185 (160, 213)
**Use of statins, n(%)**	819 (11.6)	428 (12.9)	277 (14.2)	0.003	1524 (12.4)
**Use of blood pressure lowering drugs, n(%)**	809 (11.4)	440 (13.3)	272 (14.0)	0.001	1521 (12.3)
** Abbreviations: IDU, intravenous drug user; eGFR, estimated Glomerula Filtration Rate; CKD_epi, Chronic Kidney Disease Epidemiology Collaboration*					
Chi-square or Kruskal-Wallis test as appropriate					

Supplementary Figure S1. ART-naïve PWH odds ratios of starting a 2DR vs 3DR from fitting a logistic regression model

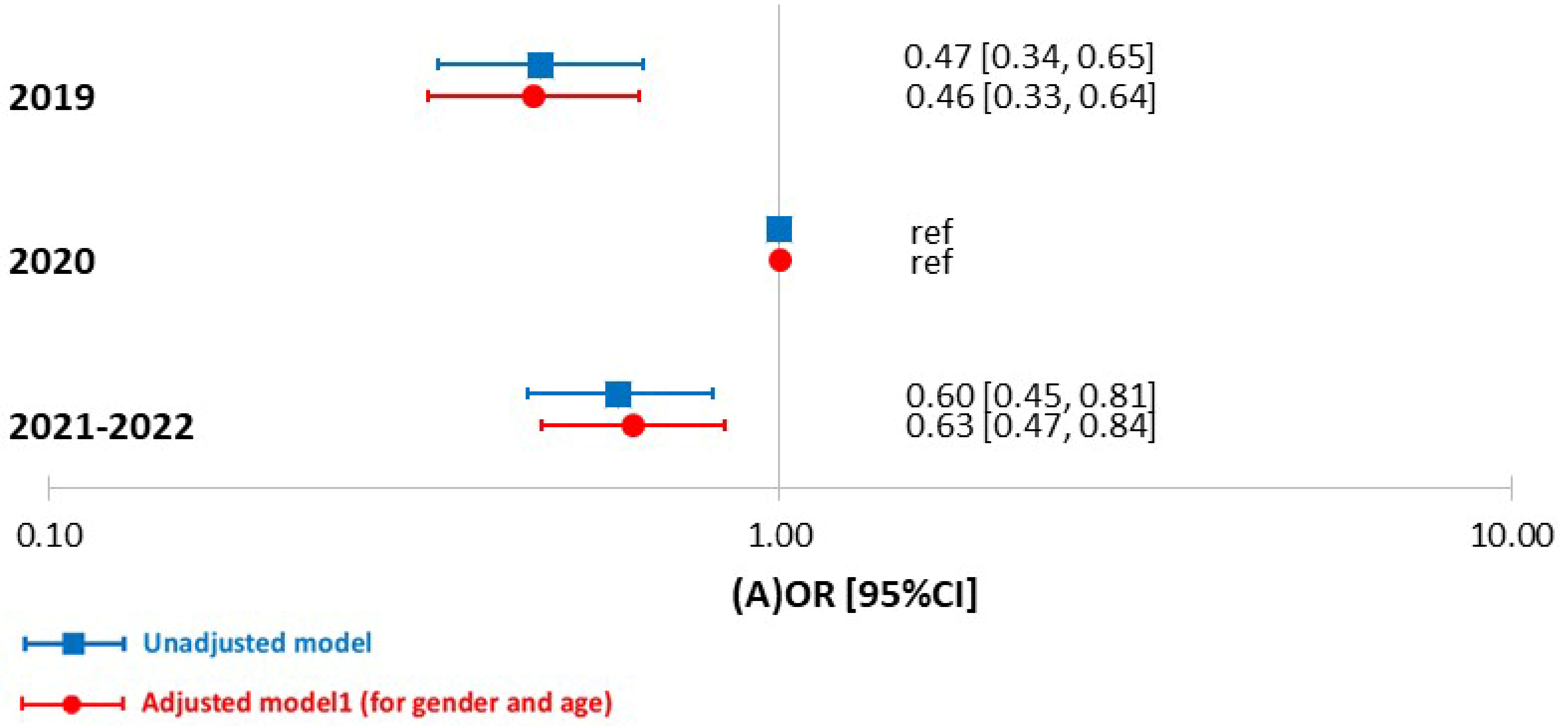

Supplementary Figure S2
ART-experienced odds ratios of starting a 2DR vs 3DR from fitting a logistic regression model.

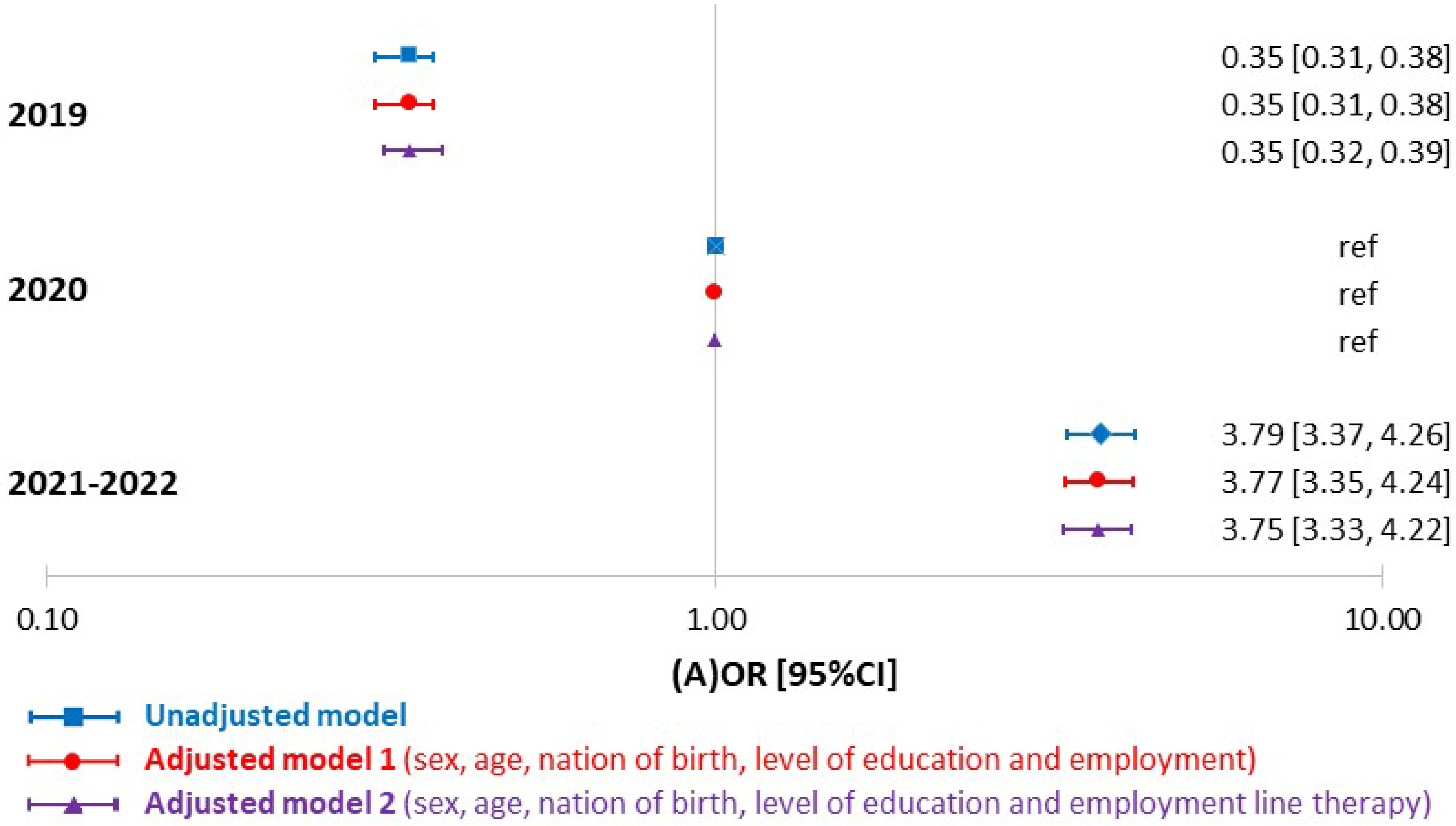

Supplementary Figure S3.
Proportions of PWH switching to 2DR 455/4465 (10%) according to calendar year in ART-experienced virologically suppressed PWH after restricting to INSTI-sparing regimens (proportion of 2DR over total number of INSTI-sparing regimens started)

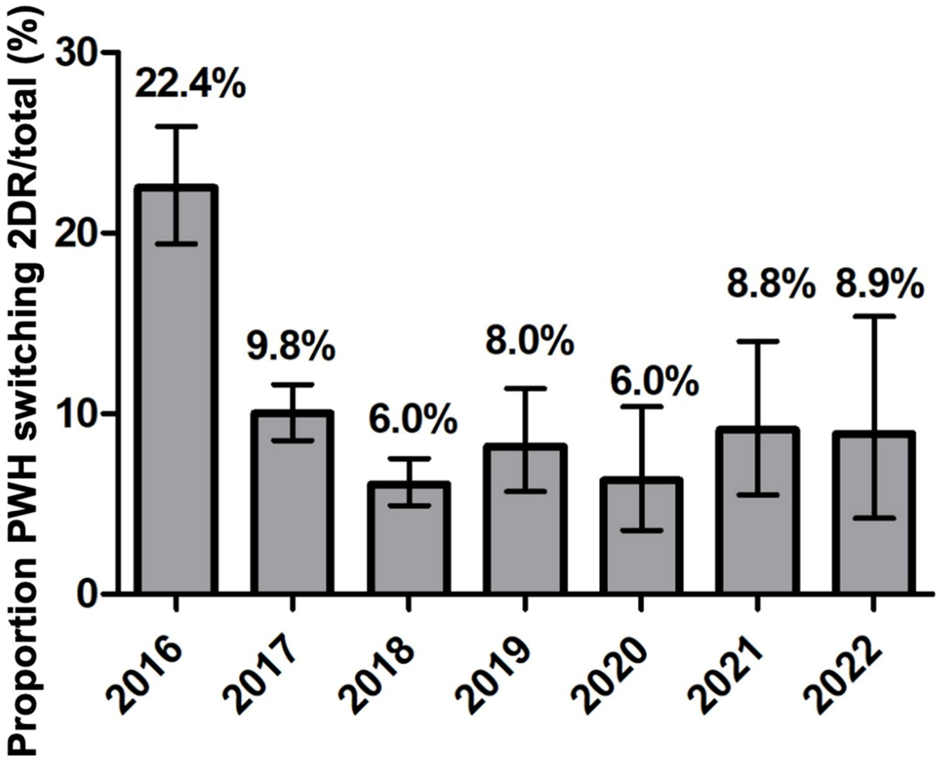

Supplementary Figure S4.
ART-experienced virologically suppressed PWH odds ratios of starting a 2DR vs 3DR from fitting a logistic regression model after restricting the analysis to INSTI-sparing regimens alone

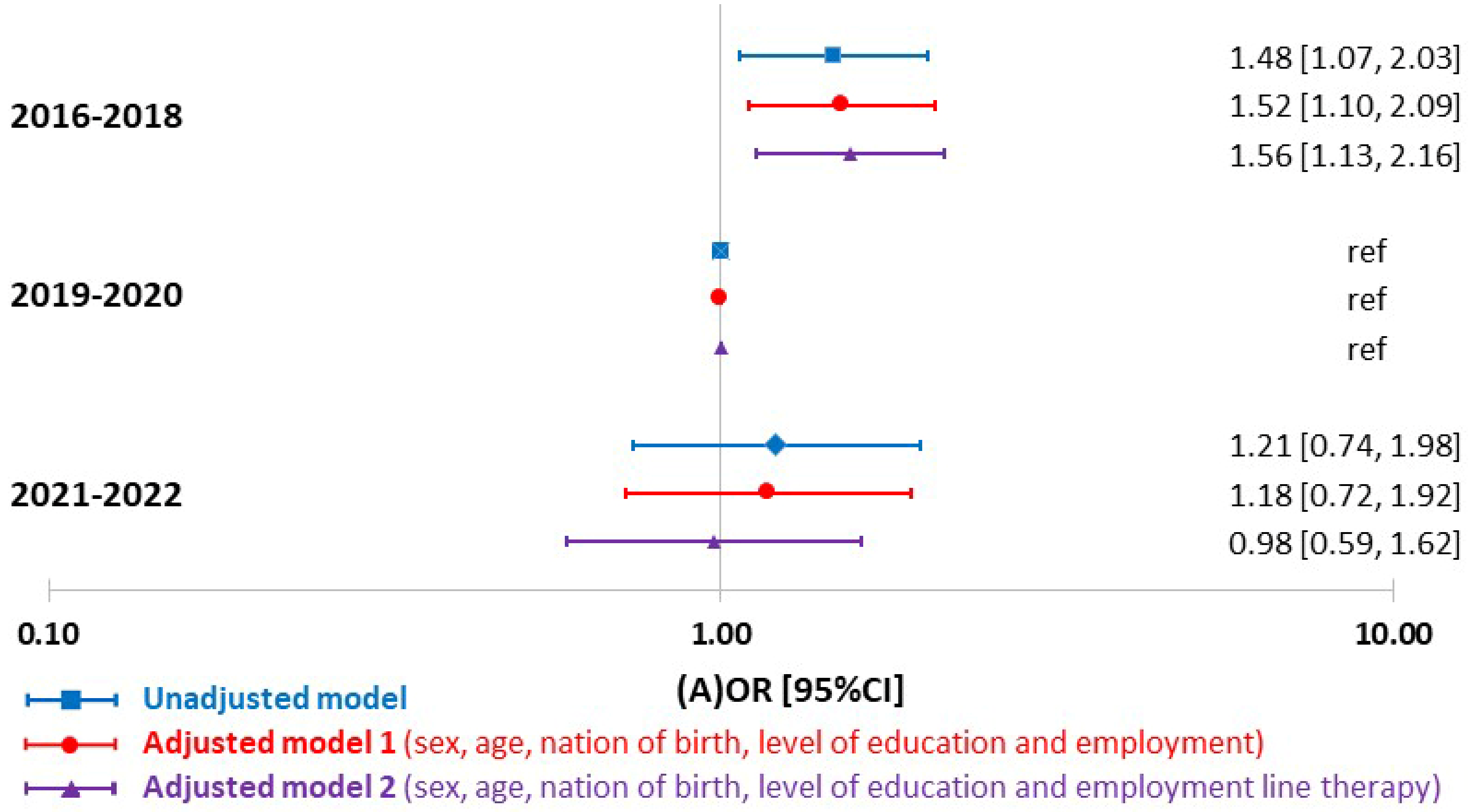

Supplement: Supplementary file 1 [file viruses-16-01822-s001.zip › viruses-3256955-supplementary.pdf]
